# Metabolism and headache

**DOI:** 10.1186/1129-2377-16-S1-A33

**Published:** 2015-09-28

**Authors:** Innocenzo Rainero

**Affiliations:** Headache Center, Department of Neuroscience, University of Turin, Turin, Italy

## Background

In the past decade, several studies have shown a significant association between migraine and several features of the metabolic syndrome (MetS), including insulin resistance, systemic hypertension, and obesity. In addition, headache is a highly prevalent symptom in several metabolic disorders. The purpose of this review was to discuss recent findings regarding the relationship between headache, migraine, and metabolic disorders.

## Methods

A computerized search of the PubMed and Cochrane Library databases was performed to identify English-language articles published between January 1, 2000, and June 30, 2015. Search terms included migraine, headache, diabetes, hypertension, obesity, and metabolic syndrome. Out of 241 articles screened, 41 were selected for review.

## Results and discussion

MetS is characterized by a cluster of metabolic abnormalities including insulin resistance, hypertension, dyslipidemia, obesity, and a proinflammatory state. Over the last two decades, different definitions and diagnostic criteria for MetS have been proposed, like the EGIR, the NCEP ATP III and the AACE criteria. Migraine, as defined by the International Headache Society (ICHD-3 beta), is a chronic neurovascular disease characterized by recurrent, disabling headache attacks. MetS and migraine are highly prevalent medical conditions, affecting 15-20% of the population. Both conditions are associated with increased risk for atherosclerotic cardiovascular disease.

The two conditions often coexist, but the pathophysiological mechanisms of this comorbidity are still under investigation. Several studies have clearly shown that insulin sensitivity is impaired in migraine, even in young, non-obese, non-diabetic, normotensive patients. Association between polymorphism of the insulin receptor gene and migraine provided inconclusive results. Prevalence and characteristics of headache in patients with diabetes have been scarcely investigated. Elevated blood pressure is a frequent clinical feature of MetS. Few studies investigated blood pressure (systolic or diastolic) abnormalities in migraine. Recently, a large demographic study found a positive association between migraine and systolic hypertension. The relationship between obesity, migraine and headache is still controversial. Weight gain is not associated with migraine prevalence but may be associated with increased headache frequency. Intriguingly, several studies suggested that neuropeptides involved in the regulations of appetite, like adiponectin, leptin, orexin-A and B, may be involved in migraine pathogenesis. In conclusion, additional research is needed to better investigate the common biological mechanisms underlying metabolic syndrome and migraine and to define the best clinical practices in patients who present with both diseases. Increased physical activity, weight loss, and management of cardiovascular risk factors may be of relevance for prevention and treatment of both conditions.

